# Prediction of inappropriate pre-hospital transfer of patients with suspected cardiovascular emergency diseases using machine learning: a retrospective observational study

**DOI:** 10.1186/s12911-023-02149-9

**Published:** 2023-04-06

**Authors:** Ji Hoon Kim, Bomgyeol Kim, Min Joung Kim, Heejung Hyun, Hyeon Chang Kim, Hyuk-Jae Chang

**Affiliations:** 1 Department of Emergency Medicine, Yonsei University College of Medicine, 50 Yonsei-ro, Seodaemun-gu, Seoul, 03722 Republic of Korea; 2 Department of Preventive Medicine, Yonsei University College of Medicine, 50 Yonsei-ro, Seodaemun-gu, Seoul, 03722 Republic of Korea; 3 AITRICS, 28 Hyoryeong-ro 77-gil, Seocho-gu, Seoul, 06627 Republic of Korea; 4 Department of Cardiology, Yonsei University College of Medicine, 50 Yonsei-ro, Seodaemun- gu, Seoul, 03722 Republic of Korea

**Keywords:** Cardiovascular emergency disease, Machine learning, Pre-hospital transfer, Inappropriate hospital

## Abstract

**Background:**

This study aimed to develop a prediction model for transferring patients to an inappropriate hospital for suspected cardiovascular emergency diseases at the pre-hospital stage, using variables obtained from an integrated nationwide dataset, and to assess the performance of this model.

**Methods:**

We integrated three nationwide datasets and developed a two-step prediction model utilizing a machine learning algorithm. Ninety-eight clinical characteristics of patients identified at the pre-hospital stage and 13 hospital components were used as input data for the model. The primary endpoint of the model was the prediction of transfer to an inappropriate hospital.

**Results:**

A total of 94,256 transferred patients in the public pre-hospital care system matched the National Emergency Department Information System data of patients with a pre-hospital cardiovascular registry created in South Korea between July 2017 and December 2018. Of these, 1,770 (6.26%) patients failed to be transferred to a capable hospital. The area under the receiver operating characteristic curve of the final predictive model was 0.813 (0.800–0.825), and the area under the receiver precision-recall curve was 0.286 (0.265–0.308).

**Conclusions:**

Our prediction model used machine learning to show favorable performance in transferring patients with suspected cardiovascular disease to a capable hospital. For our results to lead to changes in the pre-hospital care system, a digital platform for sharing real-time information should be developed.

**Supplementary Information:**

The online version contains supplementary material available at 10.1186/s12911-023-02149-9.

## Background

Cardiovascular symptoms are one of the most common reasons for patients visiting the emergency department (ED) [1]. Cardiovascular emergency conditions, such as acute myocardial infarction (AMI), are life-threatening conditions that must be recognized immediately to avoid treatment delays. Missing true cardiovascular emergencies can result in mortality and morbidity cases, which burdens healthcare services [1, 2]. Studies have reported that early recognition of cardiovascular emergency conditions at the pre-hospital stage and its rapid treatment in the hospital are crucial to improving survival rates [3–8]. Therefore, evaluating the possibility of cardiovascular emergency conditions and selecting a suitable hospital for treatment at the pre-hospital stage are critical. In this context, several studies have been conducted to predict cardiovascular emergency conditions with patient information acquired at the pre-hospital stage. However, none have focused on predicting a capable hospital for treating patients with cardiovascular emergencies [9–13]. At the pre-hospital stage, providers should select a hospital for patient transfer by sharing hospital information about patient treatment capacity while predicting cardiovascular emergencies with information obtained from patients. However, training all pre-hospital care providers to make accurate assessments is challenging. Thus, developing and validating data-based practical assessment tools at the pre-hospital stage is necessary. While efforts to predict critical events using machine learning in the medical field exist, few studies have used machine learning-based practical tools to help pre-hospital care providers decide which hospital to transfer a patient to [14–16]. Clinical tools using machine learning may prove useful at the pre-hospital stage, where advanced human resources are insufficient, rather than in hospitals, where the expertise of physicians is available.

This study was conducted to develop a prediction model using machine learning to assess the inappropriate hospital transfer of patients suspected of having cardiovascular emergency conditions at the pre-hospital stage using variables obtained from an integrated nationwide dataset.

## Methods

### Study design and setting

The present study was a retrospective observational study using three nationwide datasets from the National Fire Agency and National Emergency Medical Center in South Korea. The study protocol was approved, and informed consent was waived by the Institutional Review Board of Severance Hospital, South Korea (approval number 4-2020-0110). The present study adhered to the ethical standards of the Declaration of Helsinki.

In South Korea, the National Fire Agency, which consists of 18 provincial fire departments, oversees the public pre-hospital care system. When the pre-hospital care providers transport an emergency patient from the scene to an ED, they must complete a transfer record in compliance with field first aid standard protocol according to the National Fire Agency. This protocol covers standardized first aid procedures at the scene and guidelines for selecting a transfer hospital. This protocol is updated annually under the supervision of medical advisors. According to the Rescue and Fire Emergency Medical Service Act, all providers at the National Fire Agency are required to receive 40 h of mandatory annual training in medical skills and knowledge [17].

In South Korea, three levels of EDs are designated by the Ministry of Health and Welfare based on its human resources, emergency equipment, and availability of medical service specialists. By law, level-1 and level-2 EDs must be staffed 24 h a day with board-certified emergency physicians [18]. EDs rated at these two levels are evaluated annually by the Ministry of Health and Welfare in accordance with the Emergency Medical Service Act, to confirm whether they can provide high-level emergency medical care. The designation of levels 1 and 2 can change according to this result. In 2017, the numbers of sites for levels 1 and 2 were 36 and 119, respectively; in 2018, they were 36 and 118, respectively [19].

### Selection of participants

Patients aged > 15 years who had been transferred to EDs by the public pre-hospital care system between July 2017 and December 2018 were enrolled. Of these, patients whose Pre-Hospital Cardiovascular Registry (PHCR) had been activated were selected for inclusion, and those who could not match hospital stage information were excluded.

### Data collection and processing

The datasets were the Pre-Hospital Run Sheets (PHRS) and PHCR, both managed by the National Fire Agency; and the National Emergency Department Information System (NEDIS), operated by the National Emergency Medical Center in South Korea. The collection, processing, and integration methods of the three datasets are described in detail in Additional File 1.

### Model development

**Fig. 1:**
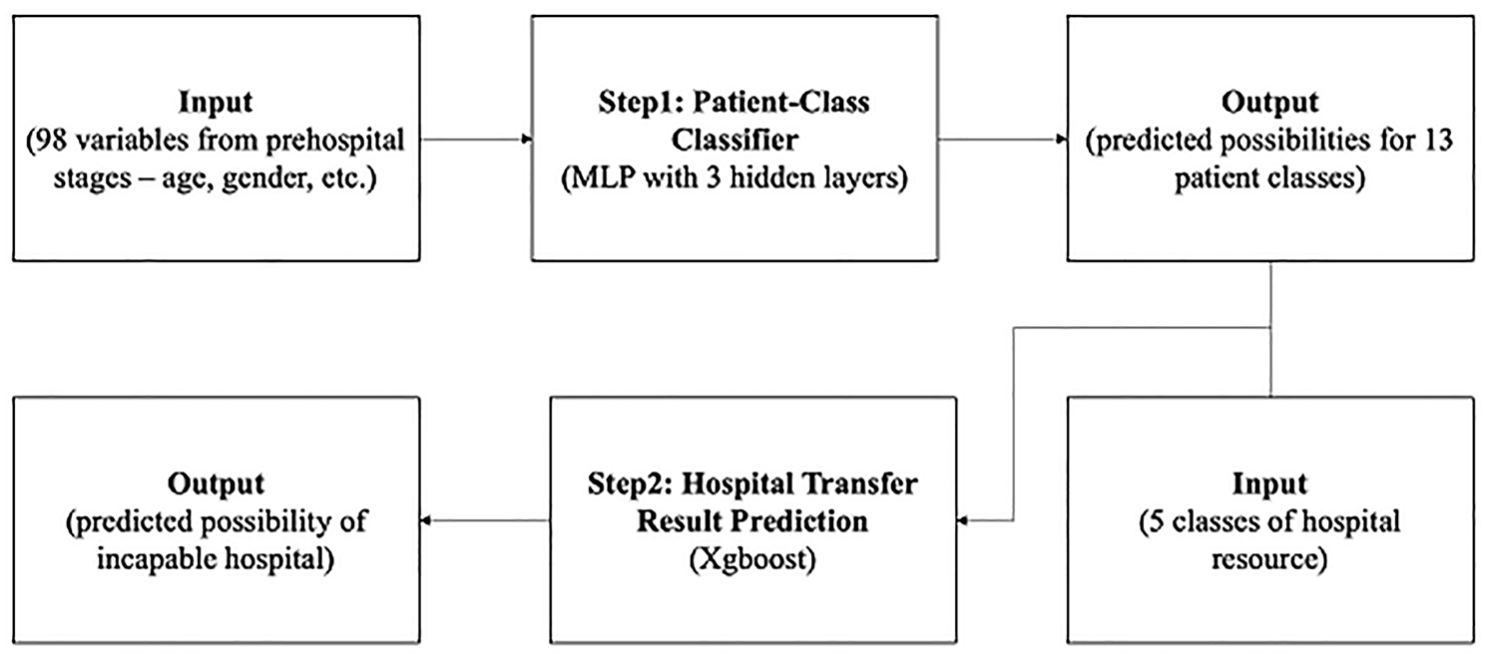
Flow chart of model development

The prediction model for transferring patients to an inappropriate hospital was developed by integrating the three datasets (PHRS, PHCR, and NEDIS). The dataset was divided into train and test sets in a 7:3 ratio, using an iterative stratification method, to ensure a similar positive ratio in every class label. The train dataset was then split into 10 folds. This model comprised two structures: the patient class prediction and the matching of hospital factors with this classification (Fig. 1). First, in the patient class prediction step, we trained a three-layer multilayer perceptron model with dropout and batch normalization using 98 variables that reflect the patient characteristics at the pre-hospital stage. Since a single patient can be categorized into multiple classes (multi-label classification problem), the loss function was constructed using each class’s binary cross-entropy loss. Accordingly, the model was designed to capture the correlation of patient class labels and simultaneously predict these correctly. Additional File 1 shows the 13 patient class labels according to the examination and treatment codes patients received in the ED. Second, for the final model to predict a patient’s transfer to an inappropriate hospital, we trained the XGBoost model. This model was developed by connecting the patient class prediction results with the hospital classification data. The model was trained to identify hospital factors that were highly associated with patients in each patient class not being transferred to an appropriate hospital. The corresponding hospital factors comprised five classes: management quality, resource availability, ED crowding, hospital occupancy, and distance between the scene and the hospital [see Additional File 1]. One of the hospital classes, resource availability, was defined as when a real-time signal of emergency resources was sent to the NEDIS from the transferred hospital. Relative crowding was defined as the number of patients in the ED compared to the average number of patients at a given time in the ED. Similar to the patient class prediction method, the final model was developed and selected by 10-fold cross-validation.

For model interpretation, we adopted the SHapley Additive exPlanation (SHAP) [20]. SHAP can explain any machine learning model’s output by calculating each feature’s impact on model prediction based on game theory. Through this process, we can understand: (1) the feature that is most important for model prediction, (2) the positive or negative direction of feature impact, and (3) the relationship between importance score (SHAP value) and feature value. Applying SHAP to our developed models, we used the Deep Explainer module, which enables the fast approximation of SHAP values in the deep learning model, and the Tree Explainer module, which optimizes the SHAP algorithm for tree ensemble methods such as XGBoost [20].

### Outcome measurement

The primary outcome was the transfer of patients to an inappropriate hospital. In the NEDIS, the disposition of patients visiting the ED was classified into four categories: admission, discharge, transfer to another hospital, and death. Transfer to an inappropriate hospital was defined as death or transfer to another hospital at the initial ED.

### Statistical analysis

All statistical analyses, model fitting, and validation were conducted using the R statistical package (www.R-project.org). The significance criterion was set as two-sided, and P values < 0.05 were considered statistically significant. Model performance was evaluated by calculating the sensitivity, specificity, accuracy, positive predictive value, negative predictive value, the area under the receiver operating characteristic curve (AUROC), and the area under the receiver precision-recall curve (AUPRC). The AUROC encompasses the area under the curve drawn with 1-specificity as the X-axis and sensitivity as the Y-axis. The AUPRC is the area under the curve summarized as the trade-off between the true positive rate and the positive predictive value for a predictive model using different probability thresholds. The AUROC and the AUPRC scores were calculated in the validation set in every epoch. We chose the model showing the highest AUROC and AUPRC scores in the validation fold. The cut-off threshold for calculating the avoidable failure of transfer to the optimal hospital was selected as the point that maximizes Youden’s J-score in the final model.

## Results

### Baseline characteristics of study participants

**Fig. 2:**
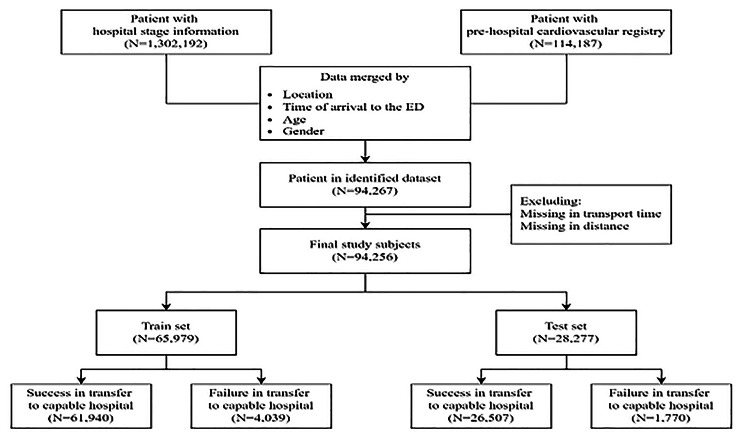
Flow chart of patient selection. (ED, emergency department)

During the study period, 94,267 public pre-hospital care system-transferred patients were matched with NEDIS data of patients created with the PHCR. Our final study participants were 94,256 patients after excluding missing data on the transport time and transfer distance to the hospital. These patients were categorized into train and test sets. Of the enrolled patients in the train set, 4,039 patients (6.1%) were transferred to an inappropriate hospital. Of the 28,277 patients enrolled in the test set, 1,770 (6.3%) failed to be transferred to an appropriate hospital (Fig. 2). The baseline characteristics of the study participants are summarized in Additional File 1.

### Model prediction

The proportions of patient classes in the test set are presented in Table 1. Table 2 shows the performance of our model that predicts 13 subclasses and transfers to an inappropriate hospital using information from the pre-hospital stage of all patients. The prediction performance rates of patient classes that had undergone cardiopulmonary resuscitation for in-hospital arrest (Class 1) or intubation (Class 2), central catheterization (Class 3), and percutaneous coronary intervention after transport (Class 5) were high: 0.865, 0.882, 0.847, and 0.929, respectively. The AUROC of the final model for predicting transfer to an inappropriate hospital was 0.813, whereas the AUPRC was 0.286.

**Table 1:** The proportions of patient classes in the test set

Prediction model	Transferred to incapable hospital (N = 1770)	Transferred to capable hospital (N = 26,507)	Total (N = 28,277)	P-value
Predicted cardiopulmonary resuscitation (Class 1)	364 (20.56)	161 (0.61)	525 (1.86)	< 0.001
Predicted intubation (Class 2)	431 (24.35)	1089 (4.11)	1520 (5.36)	< 0.001
Predicted central catheterization (Class 3)	179 (10.11)	846 (3.19)	1025 (3.62)	< 0.001
Predicted massive transfusion (Class 4)	14 (0.79)	160 (0.60)	174 (0.62)	0.413
Predicted emergency percutaneous coronary intervention (Class 5)	158 (8.93)	2356 (8.89)	2514 (8.89)	0.991
Predicted intensive care unit admission after ED process (Class 6)	133 (7.51)	4304 (16.24)	4437 (15.69)	< 0.001
Predicted emergency operation (Class 7)	123 (6.95)	191 (0.72)	314 (1.11)	< 0.001
Predicted performed magnetic resonance imaging in the ED (Class 8)	44 (2.49)	607 (2.29)	651 (2.30)	0.653
Predicted performed echocardiography in the ED (Class 9)	21 (1.19)	830 (3.13)	851 (3.01)	< 0.001
Predicted performed computed tomography angiography in the ED (Class 10)	104 (5.88)	1432 (5.40)	1536 (5.43)	0.426
Predicted psychiatric management in the ED (Class 11)	5 (0.28)	143 (0.54)	148 (0.52)	0.200
Predicted admission after ED process (Class 12)	724 (40.90)	5050 (19.05)	5774 (20.42)	< 0.001
Predicted discharge after ED process (Class 13)	364 (20.56)	161 (0.61)	11,664 (41.25)	< 0.001

Variables are expressed as counts (%); ED, emergency department

**Table 2:** The model’s performance for patient classes and transfer to an inappropriate hospital

Prediction model	Count (%)	AUROC (95% CI)	AUPRC (95% CI)	Sensitivity (95% CI)	Specificity (95% CI)	Accuracy (95% CI)	PPV (95% CI)	NPV (95% CI)
Predicted cardiopulmonary resuscitation (Class 1)	525 (1.86)	0.865 (0.845, 0.885)	0.155 (0.127, 0.189)	0.802 (0.766, 0.834)	0.757 (0.752, 0.762)	0.758 (0.753, 0.763)	0.059 (0.056, 0.061)	0.995 (0.994, 0.996)
Predicted intubation (Class 2)	1,520 (5.36)	0.882 (0.871, 0.893)	0.361 (0.338, 0.386)	0.827 (0.807, 0.845)	0.778 (0.773, 0.783)	0.781 (0.776, 0.786)	0.175 (0.170, 0.179)	0.988 (0.986, 0.989)
Predicted central catheterization (Class 3)	1,025 (3.62)	0.847 (0.832, 0.862)	0.193 (0.170, 0.219)	0.789 (0.763, 0.813)	0.760 (0.755, 0.765)	0.761 (0.756, 0.766)	0.110 (0.106, 0.114)	0.990 (0.988, 0.991)
Predicted massive transfusion (Class 4)	174 (0.62)	0.740 (0.697, 0.782)	0.021 (0.007, 0.056)	0.724 (0.653, 0.785)	0.640 (0.634, 0.646)	0.641 (0.635, 0.646)	0.012 (0.011, 0.013)	0.997 (0.997, 0.998)
Predicted emergency percutaneous coronary intervention (Class 5)	2,514 (8.89)	0.929 (0.922, 0.936)	0.589 (0.569, 0.608)	0.902 (0.889, 0.913)	0.824 (0.820, 0.829)	0.831 (0.827, 0.835)	0.334 (0.327, 0.340)	0.989 (0.987, 0.990)
Predicted intensive care unit admission after ED process (Class 6)	4,437 (15.69)	0.788 (0.779, 0.796)	0.408 (0.393, 0.422)	0.740 (0.727, 0.753)	0.697 (0.692, 0.703)	0.704 (0.699, 0.710)	0.313 (0.307, 0.319)	0.935 (0.932, 0.938)
Predicted emergency operation (Class 7)	314 (1.11)	0.706 (0.673, 0.738)	0.026 (0.013, 0.050)	0.608 (0.553, 0.661)	0.713 (0.708, 0.718)	0.712 (0.707, 0.717)	0.023 (0.021, 0.025)	0.994 (0.993, 0.995)
Predicted performed magnetic resonance imaging in the ED (Class 8)	651 (2.30)	0.816 (0.796, 0.836)	0.108 (0.086, 0.134)	0.788 (0.755, 0.818)	0.741 (0.736, 0.746)	0.742 (0.737, 0.747)	0.067 (0.064, 0.070)	0.993 (0.992, 0.994)
Predicted performed echocardiography in the ED (Class 9)	851 (3.01)	0.686 (0.666, 0.706)	0.060 (0.046, 0.078)	0.622 (0.589, 0.654)	0.658 (0.653, 0.664)	0.657 (0.652, 0.663)	0.053 (0.051, 0.056)	0.982 (0.981, 0.984)
Predicted performed computed tomography angiography in the ED (Class 10)	1,536 (5.43)	0.668 (0.653, 0.683)	0.101 (0.087, 0.117)	0.689 (0.665, 0.711)	0.557 (0.551, 0.563)	0.564 (0.558, 0.570)	0.082 (0.079, 0.085)	0.969 (0.967, 0.971)
Predicted psychiatric manage in the ED (Class 11)	148 (0.52)	0.815 (0.773, 0.857)	0.024 (0.009, 0.066)	0.831 (0.763, 0.883)	0.684 (0.678, 0.689)	0.684 (0.679, 0.690)	0.014 (0.013, 0.015)	0.999 (0.998, 0.999)
Predicted admission after ED process (Class 12)	5,774 (20.42)	0.761 (0.754, 0.769)	0.434 (0.421, 0.446)	0.696 (0.684, 0.708)	0.711 (0.705, 0.717)	0.708 (0.702, 0.713)	0.382 (0.375, 0.388)	0.901 (0.898, 0.905)
Predicted discharge after ED process (Class 13)	11,664 (41.25)	0.795 (0.790, 0.800)	0.726 (0.717, 0.734)	0.714 (0.706, 0.722)	0.726 (0.719, 0.733)	0.721 (0.716, 0.726)	0.647 (0.640, 0.653)	0.784 (0.778, 0.789)
Final model for transfer to the incapable hospital	1,770 (6.26)	0.813 (0.800, 0.825)	0.286 (0.265, 0.308)	0.739 (0.718, 0.759)	0.739 (0.733, 0.744)	0.739 (0.733, 0.744)	0.159 (0.154, 0.163)	0.977 (0.975, 0.979)

*Note.* AUROC, area under the receiver operating characteristic curve; AUPRC, area under the receiver precision-recall curve; ED, emergency department

**Fig. 3:**
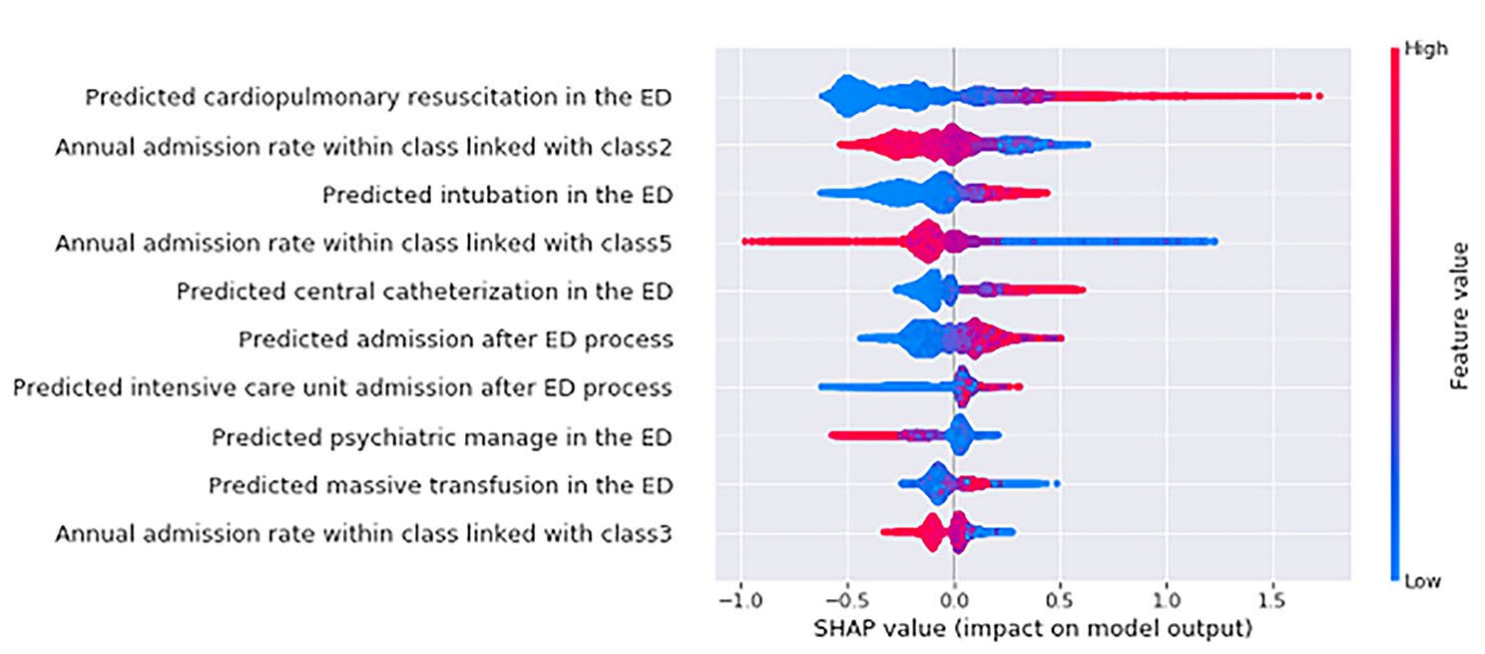
Top features in the final prediction model for transfer to inappropriate hospital. (ED, emergency department; SHAP, SHapley Additive exPlanation)

Pre-hospital stage variables with high SHAP values to predict each patient class are presented in Additional File 2. Dyspnea was included in the top ten features that predicted all classes for which urgent intervention was required, except for patients for whom coronary intervention was predicted (Class 5). Patients with less than 94% peripheral oxygen saturation at the pre-hospital stage were predicted to receive more intubation, central line catheterization, and cardiopulmonary resuscitation in the ED. TIMI scores, excepting cardiac enzyme and three-lead electrocardiogram monitoring, were high-impact features in predicting the class expected to undergo percutaneous coronary intervention (PCI) in the model. Figure 3 shows the top features in the prediction model for transfer to an inappropriate hospital. In the case of patients who were expected to receive cardiopulmonary resuscitation (Class 1), intubation (Class 2), and central line catheterization (Class 3), the probability of transfer to an inappropriate hospital was high. For patients predicted to be in classes 2, 3, and 5 according to the patient class prediction, we confirmed that if their annual hospital admission rate to their transferred hospital was high, then failure to transfer to an appropriate hospital would decrease. The model’s calibration and fairness analysis results are presented in Additional File 3.

## Discussion

Since a cardiovascular emergency condition is a life-threatening disease that causes a poor prognosis if not treated in a timely manner, a patient suspected of this condition at the pre-hospital stage should be transferred to a hospital where prompt, definite care is available [21–23]. Since it is impossible to continuously build qualified hospitals in all communities [24, 25], developing a wide-ranging system covering emergency treatment capabilities in the community is crucial [26]. Accurate distribution of patients to suitable hospitals at the pre-hospital stage is vital. When patients with cardiovascular emergency conditions are transferred to a hospital with insufficient capacity, the outcome can be fatal. Conversely, emergency resources are needlessly saturated when patients without life-threatening conditions are transferred to a higher-level hospital. Saturation of emergency resources can obstruct the immediate resuscitation of critical patients and increase the possibility of transfer to other hospitals for definite care [27–29]. Most studies that have performed cardiovascular emergency prediction based on pre-hospital settings focus on diagnosing an urgent condition or fatal disease using the patient’s features [9–13, 30]. However, the ultimate role of the pre-hospital care system is not only the prediction of a patient’s critical condition but also a timely transfer to an optimal hospital that can resolve this condition. Therefore, our model is designed to be a practical tool for pre-hospital care providers’ use in the field. Individual hospital factors should be considered in addition to patients’ features, to determine the optimal hospital at the pre-hospital stage. Our study presented a two-step model that can predict hospitals suitable for the patient class by considering hospital factors: the real-time crowding status of hospitals and EDs; distance from the scene; and treatment capacity, including PCI, of the hospital. Hospital capability can change depending on its real-time capacity or quality, even for patients with the same features; the present modeling results developed by incorporating these variable factors showed a favorable performance.

Our study results confirmed that patients in classes for which intubation or central line catheterization procedures were predicted found it more difficult to be transferred to an appropriate hospital. Interestingly, the present prediction model showed that information on the annual admission rate for the preceding year of these patient classes in the hospital to which they were transferred increased their probability of being transferred to a capable hospital. In addition, patients predicted to undergo percutaneous transluminal coronary angioplasty had a higher probability of being transferred to an appropriate hospital when annual admission rate information was combined. This is presumably because the lower ED could not supply enough medical resources for resuscitation; thus, the patient died or was transferred to another hospital for final treatment. These findings can be interpreted such that, among several hospital factors, information related to management quality for patients needing urgent intervention was the most beneficial for selecting an appropriate hospital. As the patient class prediction step in our model was designed to predict treatment to be received at the ED, rather than predicting the patient’s diagnosis, the management quality of the hospital for this treatment could be used to determine the hospital for transfer.

Our experimental modeling was developed as a practical tool for pre-hospital care providers working in the field. Pre-hospital care providers cannot manually collect and analyze on-site information in real-time to determine the hospital for transfer, considering a patient’s condition. The prediction model’s feasibility can be guaranteed only when an algorithm can perform accurate predictions by rapidly processing real-time unstructured data that are automatically collected from the pre-hospital field [31]. To identify a hospital for transfer, a shared platform for hospital information on management quality provided by various medical institutions to pre-hospital care providers is needed. Therefore, a machine learning model that can rapidly extract prediction values by inputting multitudes of information should be loaded on the digital platform.

Although various clinically useful prediction tools developed using machine learning have been introduced, the lack of explanation, the so-called black box, is a factor that makes clinical application difficult [32, 33]. Our study attempted to verify the clinical relevance of the model using explainable machine learning called SHAP and analyze the evidence for its outcome clinically. Through SHAP, we identified clinical variables from the pre-hospital stage that play an important role in predicting patient class and hospital factors that influence the final model. These explanations can convince pre-hospital care providers to use this prediction model developed by machine learning. In addition, the XGBoost used in our study can reportedly perform better in classification and regression problems involving tabular data organized as rows and columns, which are the most common data types in traditional statistical modeling [34, 35]. As the datasets used in this study consist of tubular data, we used XGBoost as an algorithm for the prediction model.

Our study has several limitations that should be considered when interpreting its findings. Since our prediction model was developed based on a retrospective observational design, a potential bias is possible. Particularly, patients who would have died at any hospital or patients who were transferred for palliative care were also defined as inappropriate transfers, which lack clinical relevance. Our study dataset and design precluded the identification of these cases. Next, although we used three prospectively collected nationwide datasets, these were not completely merged because their matching keys could not be used to ensure anonymity. As this study was performed on a fire-department-based pre-hospital care system in South Korea, the prediction model was not tested according to a specific period or geographical area, and its generalizability is not guaranteed. Finally, our prediction model has not been validated in real-world scenarios. Therefore, its usefulness should be validated through additional prospective studies in the real pre-hospital care system.

## Conclusions

Our prediction model using machine learning showed favorable performance in transferring patients with suspected cardiovascular disease to an appropriate hospital. For our results to be used as a basis to improve the pre-hospital care system in the real world, a digital platform for sharing real-time information should be developed, and additional prospective studies on its use are warranted.

## Electronic supplementary material

Below is the link to the electronic supplementary material.

Supplementary Material 1

Supplementary Material 2

Supplementary Material 3

## Data Availability

The datasets generated during the current study are available from the corresponding author upon reasonable request and with permission of National Fire Agency and National Emergency Medical Center in South Korea.

## Abbreviations

AMI: Acute Myocardial Infarction
AUPRC: Area Under the Receiver Precision-Recall Curve
AUROC: Area Under the Receiver Operating Characteristic Curve
EDs: Emergency Departments
EMTs: Emergency Medical Technicians
NEDIS: National Emergency Department Information System
PCI: Percutaneous Coronary Intervention
PHCR: Pre-Hospital Cardiovascular Registry
PHRS: Pre-Hospital Run Sheets
SHAP: SHapley Additive explanation
TIMI: Thrombolysis in Myocardial Infarction
