# Supplementary material for: Prediction of inappropriate pre-hospital transfer of patients with suspected cardiovascular emergency diseases using machine learning: a retrospective observational study
Source: BMC Med Inform Decis Mak. 2023 Apr 6;23:56. doi: 10.1186/s12911-023-02149-9 (PMC10080868; doi:10.1186/s12911-023-02149-9)
Supplement: Supplementary file 1 — Supplementary Material 1 [file 12911_2023_2149_MOESM1_ESM.docx]

- **File name:** Additional File 1
- **File format:** Microsoft Word Document (.docx)
- **Title of data:** Table 1, Table 2, and Table 3
- **Description of data:** Patient class and definition, the components of hospital classes and matched patient classes, the baseline characteristics of the study participants (n=94,256), and detailed explanation

**Table 1.** Patient class and definition

| Stage | Class | Definition |
| --- | --- | --- |
| Resuscitation | Class 1 | Predicted cardiopulmonary resuscitation in the ED^1^ |
| Resuscitation | Class 2 | Predicted intubation in the ED |
| Resuscitation | Class 3 | Predicted central catheterization in the ED |
| Resuscitation | Class 4 | Predicted massive transfusion in the ED |
| Emergency intervention | Class 5 | Predicted emergency percutaneous coronary intervention |
| Special admission | Class 6 | Predicted intensive care unit admission after ED process |
| Emergency intervention | Class 7 | Predicted emergency operation |
| Special evaluation | Class 8 | Predicted performed magnetic resonance imaging in the ED |
| Special evaluation | Class 9 | Predicted performed echocardiography in the ED |
| Special evaluation | Class 10 | Predicted performed computed tomography angiography in the ED |
| Special evaluation | Class 11 | Predicted psychiatric manage in the ED |
| Not included ^2^ | Class 12 | Predicted admission after ED process |
| Not included | Class 13 | Predicted discharge after ED process |

^1^ ED: emergency department

^2^ : Patients who did not fit into any of these four stages were labeled either needing to be hospitalized or discharged

Details on model specifications for the multilayer perceptron model are presented below. The final selected hyperparameters are indicated in bold text.

- - Model architecture (3-layer MLP)
  - input: 98
  - hidden layer1: Dense (64) + BN + elu
  - hidden layer2: Dense (128) + BN + elu + dropout (0.25)
  - hidden layer3: dense (256) + BN + elu + dropout (0.5)
  - output: Dense (13) + sigmoid → multi-label binary classification
- hparams search space
  - batch size: [2048, **1024**, 512]
  - initial lr: [1e-2, 5e-3, 1e-3, 5e-4, **1e-4**] / lr scheduler: cosine annealing
  - optimizer: [SGD, **Adam**]
  - num_epochs: [200, **300**]
  - momentum: 0.9 / weight decay: 1e-4

**Table 2.** The components of hospital classes and matched patient classes

| Class | Component | Matched patient classes |
| --- | --- | --- |
| Quality | Annual mortality within matched patients’ classes | Classes 1–7 |
|  | Annual admission rate within matched patient classes | Classes 1–7 |
| Availability^1^ | Availability for percutaneous coronary intervention | Class 5 |
|  | Availability for echocardiography | Class 9 |
|  | Availability for computed tomography angiography | Class 10 |
|  | Availability for magnetic resonance imaging | Class 8 |
|  | Availability for psychiatric treatment | Class 11 |
|  | Availability for emergency operation | Class 7 |
| ED crowding | ED bed occupancy^2^ | All classes |
|  | Relative crowding ^3^ | All classes |
| Hospital occupancy | Hospital bed occupancy^2^ | All classes |
|  | Available bed in intensive care unit | All classes |
| Distance | Distance from scene to hospital | All classes |

^1^ Availability: represents whether a specific treatment can be provided at the time of transfer. EDs in South Korea are required to send information on real-time availability for specific treatment to the NEMC, and we developed our final model using this dataset.

^2^ Bed occupancy: the number of available beds/total number of beds

^3^ Relative crowding: the current number of patients/the average number of patients simultaneously in the ED

Details on model specifications for the XGBoost model are as follows. The final selected hyperparameters are indicated in bold text.

- hparams search space
  - eta: [0.1, 0.05, **0.01**, 0.001]
  - max depth: [2,3,4,**5**,8]
  - num_rounds: [200,500,**1000**,10000]
  - tree methods: [’auto’, **‘gpu_hist’**]

**Table 3.** Baseline characteristics of the study participants (n=94256)

| Variables | N / Mean | % / ± SD^1^ | | | |
| --- | --- | --- | --- | --- | --- |
| Transferred to an incapable hospital |  |  | | | |
| No | 88447 | 93.8 | | | |
| Yes | 5809 | 6.2 | | | |
| Sex |  |  | | | |
| Female | 40632 | 43.1 | | | |
| Male | 53624 | 56.9 | | | |
| Age, years | 61.7 | 19.2 | | | |
| *Patient’s symptoms* |  |  | | | |
| Chest pain, n (%) | 34384 | 36.5 | | | |
| Dyspnea, n (%) | 50681 | 53.8 | | | |
| Palpitation, n (%) | 6682 | 7.1 | | | |
| Chest discomfort, n (%) | 1306 | 1.4 | | | |
| Syncope, n (%) | 15670 | 16.6 | | | |
| Nausea, n (%) | 4433 | 4.7 | | | |
| Emesis, n (%) | 3446 | 3.7 | | | |
| Dizziness, n (%) | 9096 | 9.7 | | | |
| Cold sweating, n (%) | 9078 | 9.6 | | | |
| Consciousness fluctuation, n (%) | 3673 | 3.9 | | | |
| *Past medical history* |  |  | | | |
| Hypertension, n (%) | 32461 | 34.4 | | | |
| Diabetes, n (%) | 17335 | 18.4 | | | |
| Cerebral vascular disease, n (%) | 5267 | 5.6 | | | |
| Lung disease, n (%) | 10088 | 10.7 | | | |
| Cardiovascular disease, n (%) | 24871 | 26.4 | | | |
| Tuberculosis, n (%) | 345 | 0.4 | | | |
| Hepatitis, n (%) | 244 | 0.3 | | | |
| Liver cirrhosis, n (%) | 796 | 0.8 | | | |
| Allergic disease, n (%) | 264 | 0.3 | | | |
| Cancer, n (%) | 6939 | 7.4 | | | |
| Kidney disease, n (%) | 3234 | 3.4 | | | |
| *Levels of consciousness* |  |  | | | |
| A (Alert) | 86235 | 91.5 | | | |
| V (Verbal Stimuli) | 4468 | 4.7 | | | |
| P (Response to pain/pressure) | 2653 | 2.8 | | | |
| U (Unresponsive to stimuli) | 900 | 1.0 | | | |
| *Vital signs* |  |  | | | |
| Diastolic blood pressure |  |  | | | |
| 60–90 | 65877 | 69.9 | | | |
| <60 | 5429 | 5.8 | | | |
| >90 | 22950 | 24.3 | | | |
| Systolic blood pressure |  |  | | | |
| 90–140 | 62040 | 65.8 | | | |
| <90 | 3268 | 3.5 | | | |
| >140 | 28948 | 30.7 | | | |
| Pulse rate |  |  | | | |
| 60–100 | 59865 | 63.5 | | | |
| <60 | 5245 | 5.6 | | | |
| >100 | 29146 | 30.9 | | | |
| Respiratory rate |  |  | | |  |
| 12–20 | 71279 | 75.6 | | |  |
| <12 | 770 | 0.8 | | |  |
| >20 | 22207 | 23.6 | | |  |
| Body temperature |  |  | | |  |
| 35.5–36.6 | 45277 | 48 | | |  |
| <35.5 | 1539 | 1.6 | | |  |
| >36.5 | 47440 | 50.3 | | |  |
| Peripheral oxygen saturation |  |  | | |  |
| 94+ | 79672 | 84.5 | | |  |
| <94 | 14584 | 15.5 | | |  |
| Blood sugar level |  |  | | |  |
| 70+ | 93460 | 99.2 | | |  |
| <70 | 796 | 0.8 | | |  |
| *Location of Chest Pain* |  |  | | |  |
| Left, n (%) | 11287 | 12.0 | | |  |
| Right, n (%) | 3021 | 3.2 | | |  |
| Substernal, n (%) | 6102 | 6.5 | | |  |
| Pit of the stomach (anticardium), n (%) | 11821 | 12.5 | | |  |
| Other, n (%) | 3108 | 3.3 | | |  |
| *Type of Chest Pain* |  |  | | |  |
| Pressing pain, n (%) | 4729 | 5.0 | | |  |
| Tightening/squeezing pain, n (%) | 11377 | 12.1 | | |  |
| *Type of Chest Pain* |  | |  | |  |
| Bursting pain, n (%) | 1143 | | 1.2 | |  |
| Heaviness, n (%) | 11552 | | 12.3 | |  |
| Ripping pain, n (%) | 1560 | | 1.7 | |  |
| Other, n (%) | 5235 | | 5.6 | |  |
| *Radiating Pain of Chest Pain* |  | |  | |  |
| Left arm, n (%) | 2238 | | 2.4 | |  |
| Right arm, n (%) | 1067 | | 1.1 | |  |
| Back, n (%) | 2910 | | 3.1 | |  |
| Neck, n (%) | 1579 | | 1.7 | |  |
| Other, n (%) | 1440 | | 1.5 | |  |
| *Factors Exacerbating Chest Pain* |  | |  | |  |
| Exercise, n (%) | 3217 | | 3.4 | |  |
| Other, n (%) | 1071 | | 1.1 | |  |
| *Factors Relieving Chest Pain* |  | |  | |  |
| Nitroglycerin, n (%) | 4445 | | 4.7 | |  |
| Rest, n (%) | 4525 | | 4.8 | |  |
| Other, n (%) | 946 | | 1.0 | |  |
| Pain score (0–10) | 1.8 | | 3.0 | |  |
| *Duration of Chest Pain* |  | |  | |  |
| No chest pain |  | |  | |  |
| Less than 5 minutes | 4942 | | 5.2 | |  |
| 5–20 minutes | 8038 | | 8.5 | |  |
| 20 minutes or more | 18871 | | 20.0 | |  |
| Unknown | 2533 | | 2.7 | |  |
| Transport time, minutes | 12.7 | | | 38.8 |  |
| Nitroglycerin try in pre-hospital stage, n (%) | 5849 | | | 6.2 |  |
| Sildenafil citrate try in pre-hospital stage, n (%) | 71 | | | 0.1 |  |
| 3-lead electrocardiogram apply, n (%) | 35568 | | | 37.7 |  |
| 12-lead electrocardiogram apply, n (%) | 7899 | | | 8.4 |  |
| Normal sinus rhythm on 3-lead electrocardiogram, n (%) | 18984 | | | 20.1 |  |
| Sinus tachycardia on 3-lead electrocardiogram, n (%) | 5128 | | | 5.4 |  |
| Regular wide QRS tachycardia on 3-lead  electrocardiogram, n (%) | 306 | | | 0.3 |  |
| Irregular wide QRS tachycardia on 3-lead  electrocardiogram, n (%) | 92 | | | 0.1 |  |
| Regular narrow QRS tachycardia on 3-lead  electrocardiogram, n (%) | 932 | | | 1.0 |  |
| Irregular narrow QRS tachycardia on 3-lead  electrocardiogram, n (%) | 436 | | | 0.5 |  |
| Sinus bradycardia on 3-lead electrocardiogram, n (%) | 1909 | | | 2.0 |  |
| Second-degree atrioventricular block on 3-lead  electrocardiogram, n (%) | 118 | | | 0.1 |  |
| Third-degree atrioventricular block on 3-lead  electrocardiogram, n (%) | 64 | | | 0.1 |  |
| Other atrioventricular block on 3-lead  electrocardiogram, n (%) | 199 | | | 0.2 |  |
| Unrecognizable on 3-lead electrocardiogram, n (%) | 7400 | | | 7.9 |  |
| ST segment elevation on 12-lead electrocardiogram, n (%) | 1572 | | | 1.7 |  |
| *Circumstances of Cardiovascular Disease Occurrence* | | | |  |  |
| Daily life | 65824 | | | 69.8 |  |
| While sleeping/resting | 13848 | | | 14.7 |  |
| Working | 2759 | | | 2.9 |  |
| Sports/leisure | 1101 | | | 1.2 |  |
| Education/training | 227 | | | 0.2 |  |
| On the move | 4644 | | | 4.9 |  |
| Under treatment | 998 | | | 1.1 |  |
| Other | 4855 | | | 5.2 |  |
| Thrombolysis in myocardial infarction risk score except for cardiac enzyme | 1.1 | | | 0.9 |  |

^1^ SD: Standard deviation.

**The collection, processing, and integration methods of the three datasets**

PHRS are electronically filled out to record basic pre-hospital operation information in the National Fire Agency. Patients’ age, sex, past medical history, consciousness level, and vital signs were extracted from PHRS. Vital sign measurement in a pre-hospital environment is prone to errors such as equipment failure and human error in digitalizing the data from manual notes. Therefore, we converted these to categorical variables to encode meaningful information and reduce negative effects from outliers. In cases where patients’ symptoms recorded in the PHRS include chest pain, dyspnea, palpitations, syncope, or other suspected cardiovascular events, care providers are additionally required to enter information into the PHCR for cardiovascular emergency diseases screening. PHCR consists of a two-step systematic entry. First, chief complaints, accompanying symptoms, and the onset of symptoms, as well as the location, characteristic, intensity, radiation, duration, aggravating and relieving factors of chest pain, are evaluated. Based on this information, care providers must record whether the patient is predicted to have a cardiovascular emergency diagnosis in the hospital. In such cases, additional patient information should be obtained and recorded. This should include the following: response of sublingual nitroglycerin, 3-lead and 12-lead electrocardiographic findings, and thrombolysis in myocardial infarction risk (TIMI) score based on the exclusion of cardiac enzyme marker results. Since 2013, the PHCR has been amended four times by the Expert Quality Management Committee. Based on data from this registry, a pre-hospital quality management program is underway.

NEDIS is a nationwide computerized system used to collect and analyze medical information of patients who visit EDs in South Korea. This dataset includes several types of information, such as emergency care, undergone procedures, and clinical outcomes, of patients in the hospital phase. NEDIS provides real-time information of emergency resource availability. This registry is managed according to the standardized protocol distributed by the National Emergency Medical Center. Moreover, since its establishment in 2003, it has been revised several times and is now updated to version 3.2. Age, sex, location of the patient, and the arrival time to the ED (±10 min) were the matching variables between the databases from the National Fire Agency and NEDIS.
