# Supplementary material for: Prediction of inappropriate pre-hospital transfer of patients with suspected cardiovascular emergency diseases using machine learning: a retrospective observational study
Source: BMC Med Inform Decis Mak. 2023 Apr 6;23:56. doi: 10.1186/s12911-023-02149-9 (PMC10080868; doi:10.1186/s12911-023-02149-9)

- **File name:** Additional File 2
- **File format:** Microsoft Word Document (.docx)
- **Title of data:** Figure 1
- **Description of data:** Pre-hospital variables with high SHapley Additive exPlanation (SHAP) values to predict each patient class

**Figure 1.** Pre-hospital variables with high SHapley Additive exPlanation (SHAP) values to predict each patient class


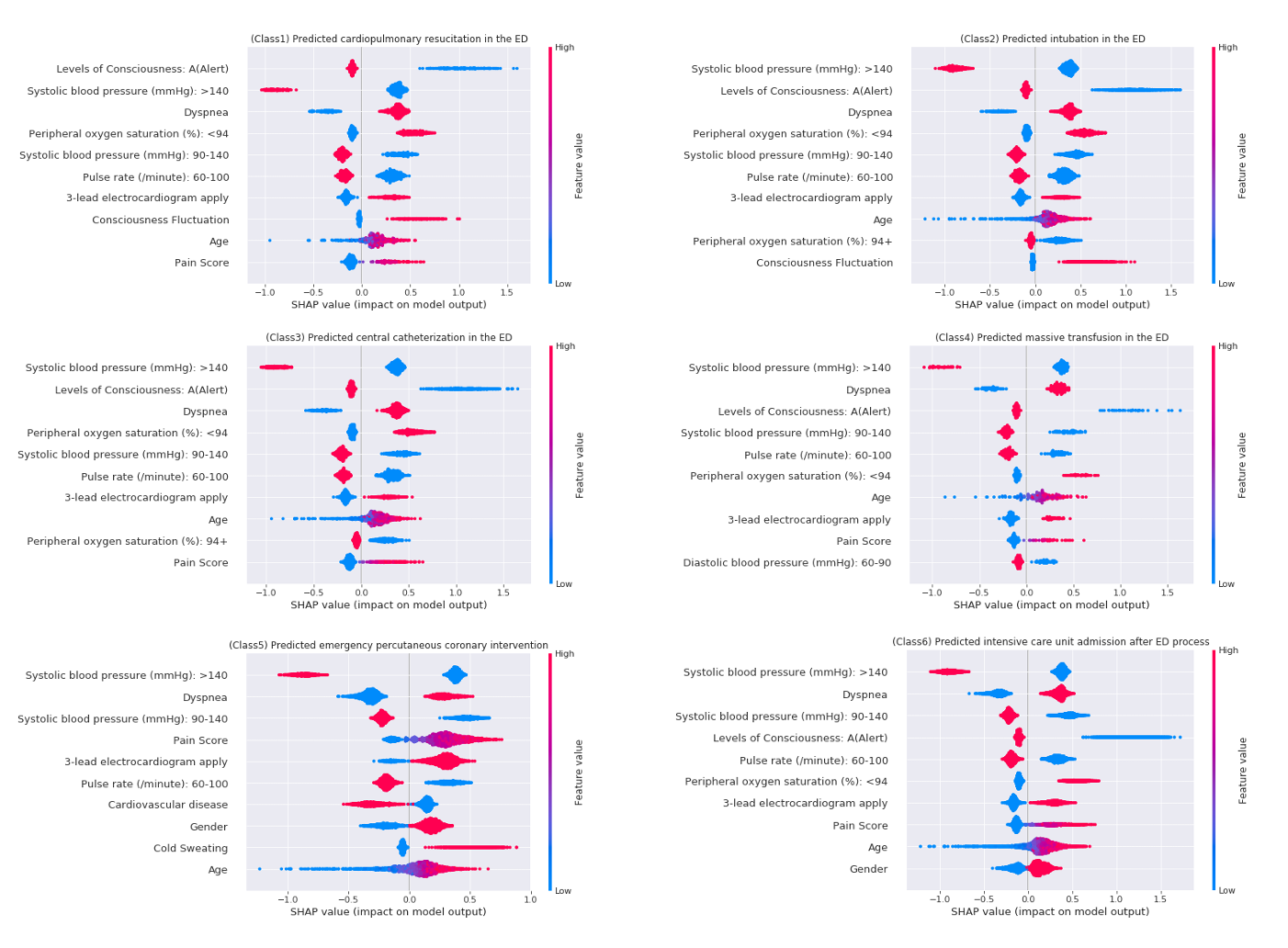


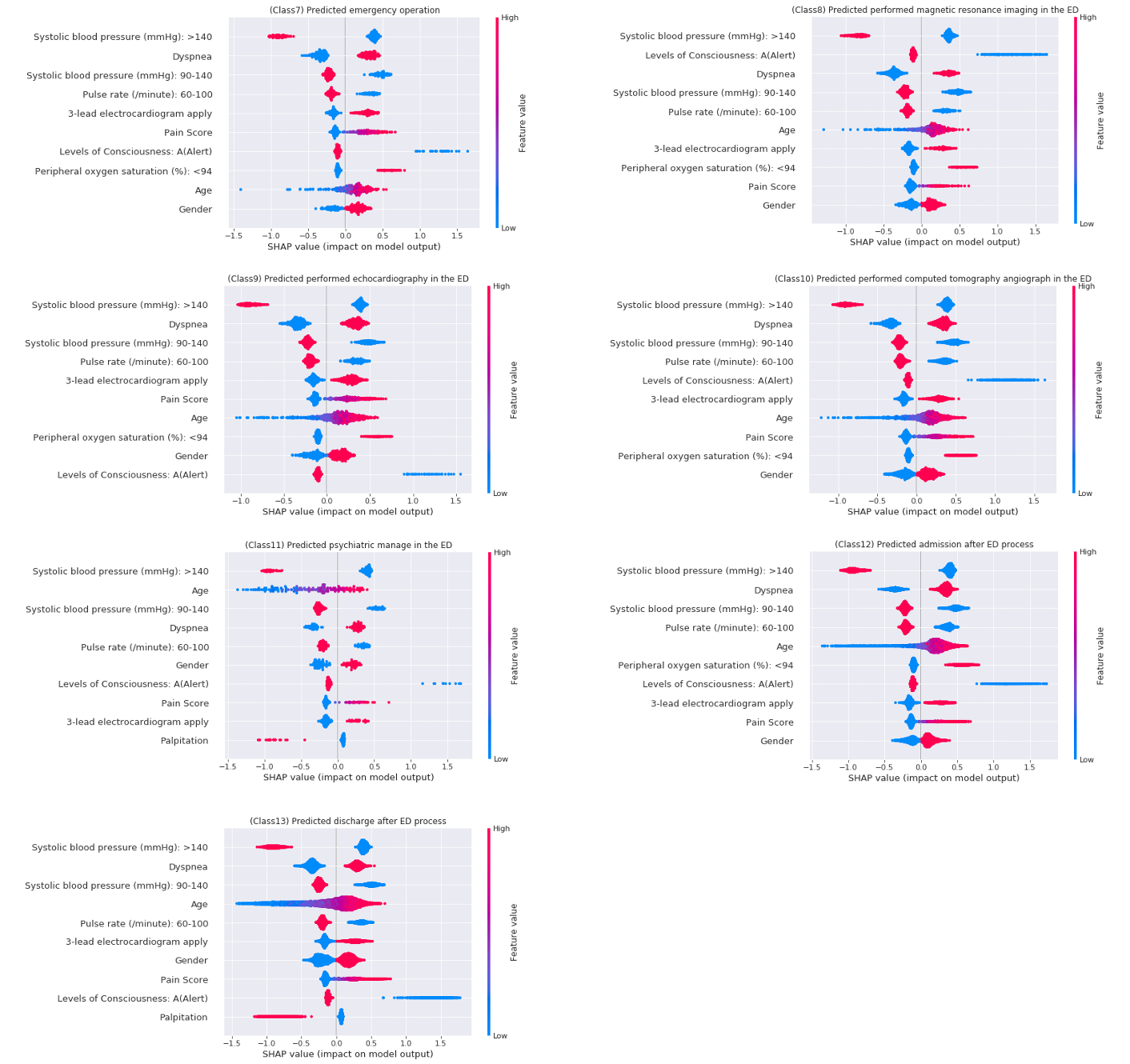

Supplement: Supplementary file 2 — Supplementary Material 2 [file 12911_2023_2149_MOESM2_ESM.docx]
