# Supplementary material for: Prediction of inappropriate pre-hospital transfer of patients with suspected cardiovascular emergency diseases using machine learning: a retrospective observational study
Source: BMC Med Inform Decis Mak. 2023 Apr 6;23:56. doi: 10.1186/s12911-023-02149-9 (PMC10080868; doi:10.1186/s12911-023-02149-9)

- **File name:** Additional File 3
- **File format:** Microsoft Word Document (.docx)
- **Title of data:** Additional analysis

**Description of data**: confusion matrix, fairness analysis and calibration characteristics of modeling, Calibration characteristics of modelling

**Confusion matrix**


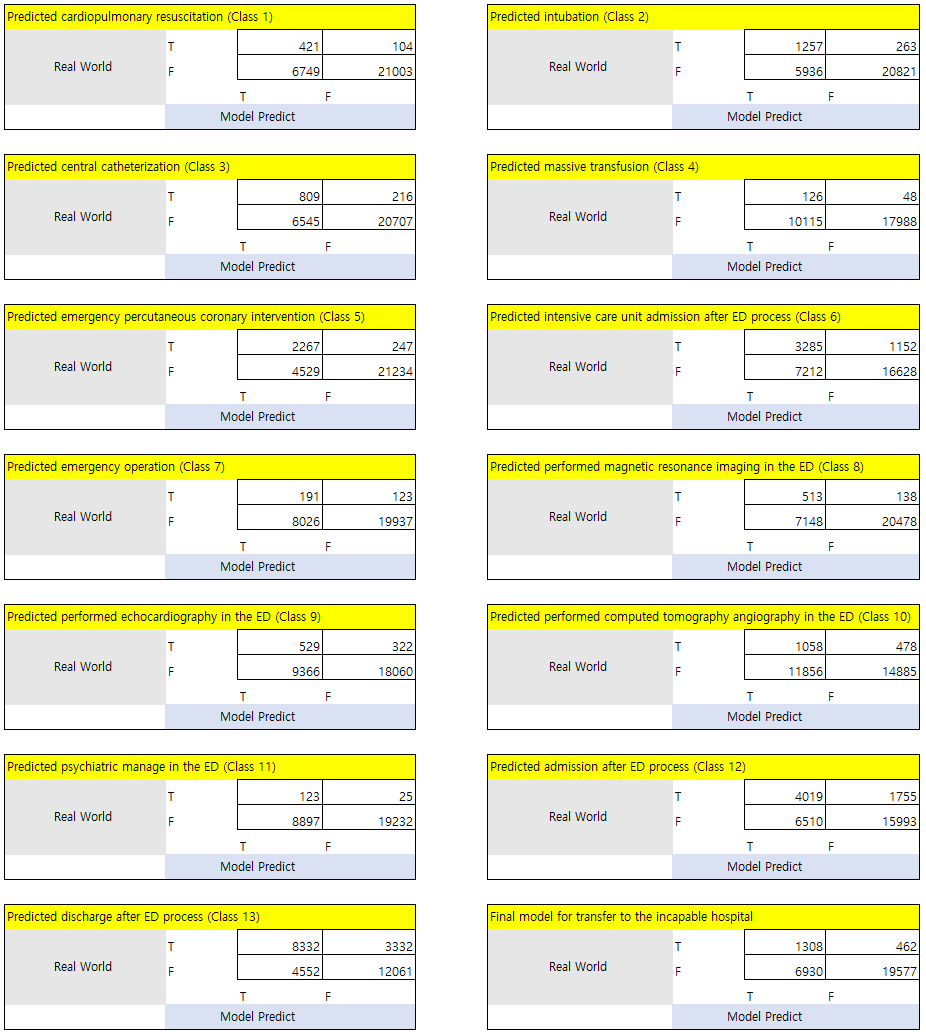


**Fairness analysis according to gender and age**


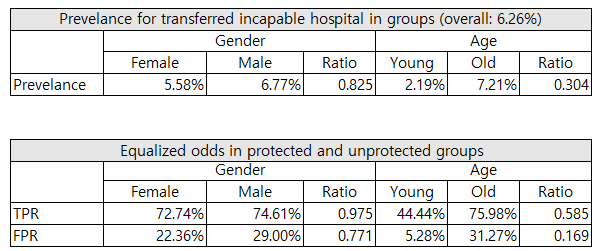


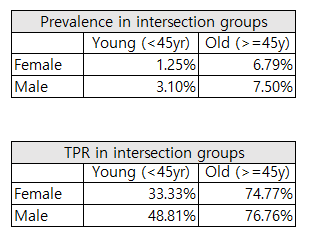


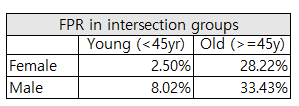


**Calibration characteristics of modelling**


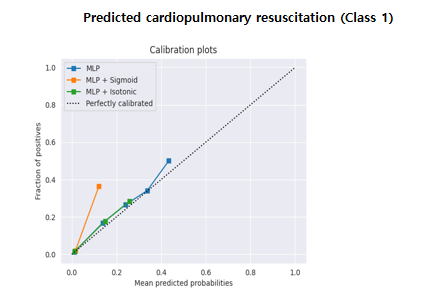

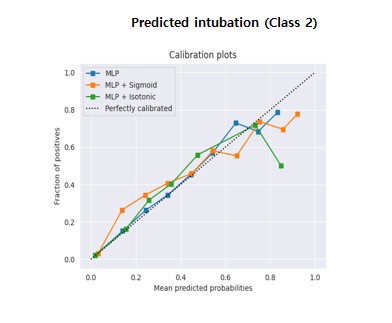

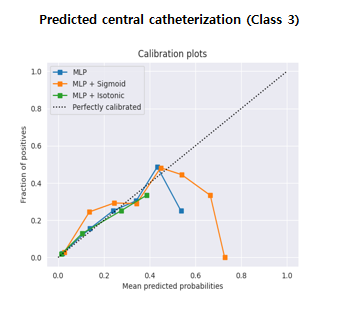

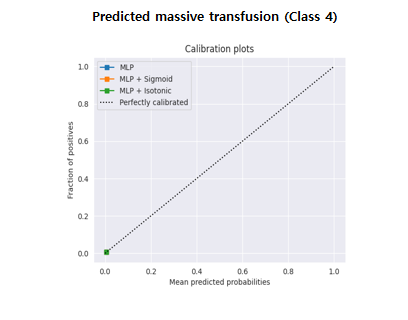


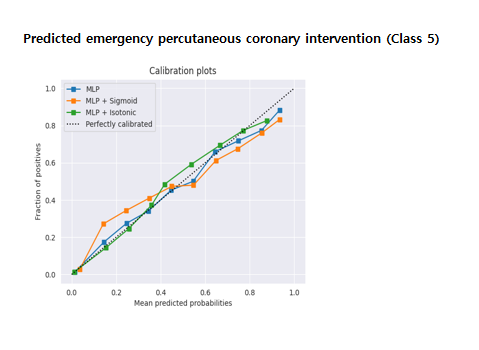

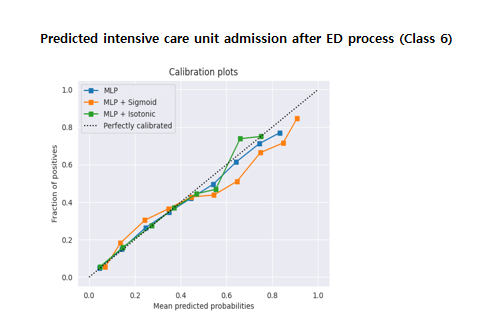

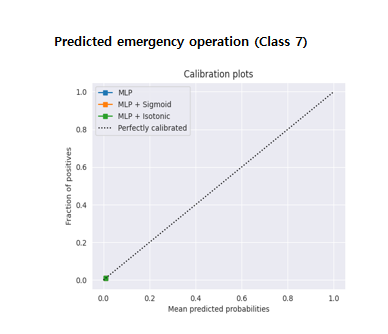

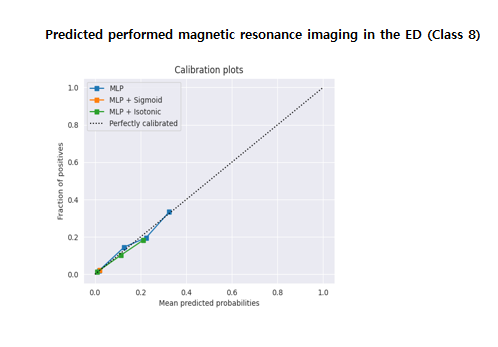


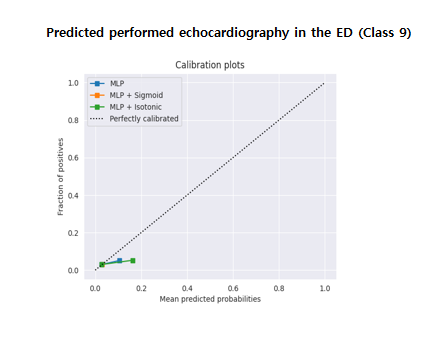

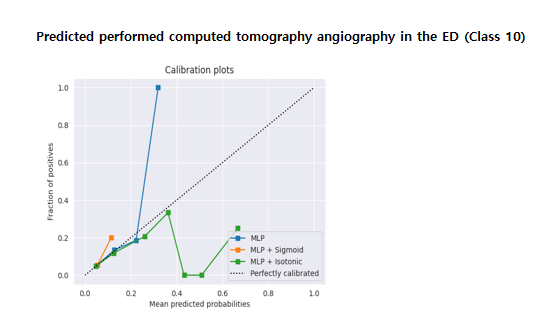


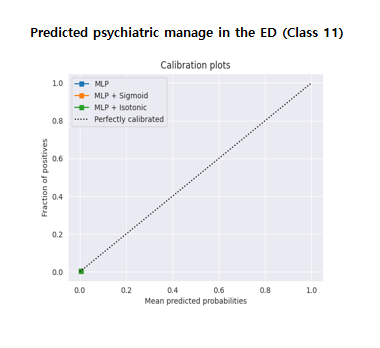

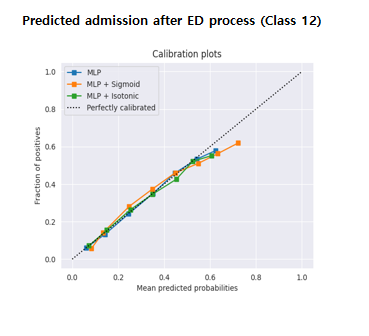

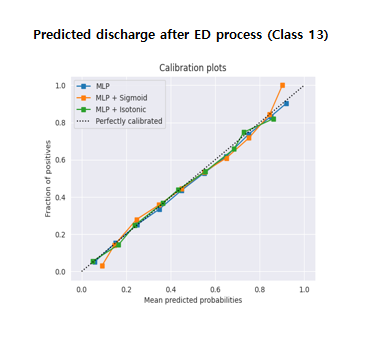

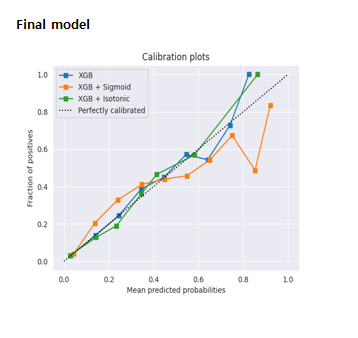

Supplement: Supplementary file 3 — Supplementary Material 3 [file 12911_2023_2149_MOESM3_ESM.docx]
